# Supporting Self-Employed Cancer Survivors to Continue Working: Experiences of Social Welfare Counsellors and Survivors

**DOI:** 10.3390/ijerph18084164

**Published:** 2021-04-15

**Authors:** Steffen Torp, Birgit Brusletto, Bente Nygaard, Tina Blomquist Withbro, Linda Sharp

**Affiliations:** 1 Department of Health, Social & Welfare Studies, University of South-Eastern Norway, PO Box 4, 3199 Borre, Norway; birgit.brusletto@usn.no (B.B.); bennyg@siv.no (B.N.); Tina.Blomquist.Withbro@nav.no (T.B.W.); 2 Population Health Sciences Institute, Newcastle University, Newcastle-upon-Tyne NE1 4LP, UK; Linda.Sharp@newcastle.ac.uk

**Keywords:** self-employment, return to work, vocational rehabilitation, social welfare, health promotion

## Abstract

Few studies have investigated the support needed or received by self-employed cancer survivors to continue working. In Norway, the Labour and Welfare Administration (NAV) is responsible for supporting people both practically and financially to continue or return to work following ill health. Social welfare counsellors (NAV counsellors) are responsible for guiding workers in their effort to return to work. This study aimed to investigate NAV counsellors’ experiences of supporting self-employed cancer survivors. We also report how self-employed people experienced the support they received from NAV during and after cancer treatment. We conducted individual in-depth interviews among seven self-employed cancer survivors and seven NAV counsellors with experience in supporting self-employed cancer survivors. The survivors experienced NAV as largely absent and considered that the support offered was not very useful. The NAV counsellors stated that self-employed workers are in a difficult situation and that regulations and means of support were primarily designed to fit salaried workers. While they felt they were supposed to function as an “employer” for the self-employed, they found this difficult because of lack of time, expertise and means for supporting self-employed. These findings suggest that the social welfare system in Norway is not adapted to support sick self-employed people appropriately.

## 1. Introduction

Earlier diagnosis and advances in treatment mean cancer survival is increasing. Nowadays, almost three-quarters of cancer patients survive five years post-diagnosis [1]. Approximately 40% of all cancer patients are of working age (20–65 years) [2] and this age-group often have the highest survival. In Europe, approximately three-quarters of people in this age-group are working (i.e., employed or self-employed) [3].

From a societal perspective, high rates of workforce participation are economically beneficial [4]. For individuals, work provides income, enhances self-esteem and provides social contact [5]. For cancer survivors, retaining or returning to work post-diagnosis is important both economically (protecting against cancer-related financial hardship, which is associated with lower quality-of-life) [6,7] and psychologically (providing “normality” in life after having experienced the trauma of cancer) [8]. However, many survivors experience adverse effects of cancer and its treatment, including physical and functional limitations, fatigue, cognitive problems and depression [9,10,11] that may hamper continuing work. Around 60–75% of survivors are employed [12,13,14], and diagnosis, treatment and socio-demographic and work-related factors influence this [12,15]. 

Although evidence is growing on the effects of cancer on work retention and on how to best support returning to work after cancer [16], almost all this research has focused on salaried workers. Few studies even mention self-employed survivors [17] and the very few that focus on this group indicate that self-employed survivors face more, and different, difficulties around work retention than salaried survivors [17,18,19,20,21,22]. What is particularly lacking is research on the support needed or received by self-employed survivors. 

In Europe, 14% of workers are self-employed [23]. This sector of the labour market is seen as a means of promoting entrepreneurship, innovation and job creation and a key enabler of sustained economic growth, well placed to reap opportunities from globalization and technological changes. Measures to encourage self-employment are being implemented widely [24]. 

The self-employed are a heterogeneous group, comprising people at both ends of the socio-economic spectrum (i.e., highly educated professionals with good incomes, such as doctors and lawyers, and less well educated people with much lower incomes, such as farmers and small shop owners). Most self-employed work on their own account, but 30% also provide jobs for other people. A significant proportion are in precarious work situations or dependent on one main client [24,25]. 

What the self-employed share is not having an employer. Self-employment offers freedom and autonomy regarding type of work, pace and scheduling [26] and self-employed people report greater job satisfaction and better quality-of-life than salaried workers [26]. However, the self-employed often have heavy workloads and work long and irregular hours [21,27]. Many report considerable stress and difficulties combining work and family life [27]. Social isolation and loneliness are common. In addition, income can be volatile [28] and, in most countries, social welfare provisions are worse for the self-employed than salaried workers [29]. Uncertainty about income when facing medical problems is a particularly important stressor for self-employed people [30]. Results of studies comparing salaried and self-employed cancer survivors post-diagnosis have shown that the self-employed suffer a bigger reduction in the number of hours worked after cancer, but still work more hours on average than salaried survivors [21] and that self-employed survivors report poorer health, quality of life and work ability [20]. 

People in Norway have in general good public rights and in no other European country are more people employed in the public sector, relatively to those employed in the private sector [3,31]. One of the One of the public sectors employing many is the Labour and Welfare Administration (NAV). NAV administers one third of Norway’s national budget through schemes such as unemployment benefit, sickness benefit, pensions and child benefit. The 2006 “NAV reform” brought together three former welfare service offices (state insurance office, state employment office and components of municipal social services) [32] to provide “seamless” support for clients. A major NAV responsibility is “activation work”: motivating, compelling and assisting citizens who are having difficulties with labour market participation. One of the aims and actions of the reform was to change the role of NAV officers from being caseworkers to being counsellors. The NAV counsellors were given discretionary powers to make judgements about clients’ ability and willingness to work, what kind of activation measures the clients would benefit from, and the benefits they may receive [33,34]. The counsellors themselves have varied backgrounds and levels of education [35,36] and they are trained in activation work and work capacity assessment [33]. 

Despite the best intentions, studies have shown that the NAV reform has not been very successful since the rate of work retention/return to work among clients has been rather low [35]. Suggested reasons for this are, among others, heavy workloads among counsellors due to large portfolios of clients [37], medical declarations without relevant information about the work capacity of clients [38], poor training in work capacity assessment and activation work [33] and poor training in use of information and communication technology tools in this work [37]. Overall, Andreassen [33] claims that there is a need to improve NAV counsellors’ knowledge base for professional discretionary reasoning. To our knowledge, no studies have explicitly investigated the services NAV provides for self-employed people in general or self-employed people with cancer in particular. 

Norway’s social welfare services for both salaried employees and self-employed people differ from services provided in many other countries [21]. Nevertheless, we believe that an exploration of how NAV counsellors experience supporting self-employed people and how self-employed people with cancer experience the support they receive is of wider relevance. The situations of self-employed people in general and cancer survivors in particular are similar in many countries and their particular challenges are, therefore, rather universal.

Therefore, in this study, we aimed to explore self-employed survivors’ experiences with NAV and NAV counsellors’ experiences of supporting self-employed cancer survivors in their work retention/return to work. This paper complements our previous publication in which we reported on experiences with returning to work among the same series of self-employed cancer survivors [22]. 

## 2. Materials and Methods

### 2.1. Setting 

In Norway, the employer plays an important role in supporting employees to return to, or retain, work following an absence due to illness. It is a legal requirement to offer suitable work and make any necessary adjustments the worker may require (e.g., reduced hours, changes in tasks, providing different equipment) [39]. The employer must also organize a meeting (“Dialogue meeting 1”) with the employee after four weeks of sick leave to discuss possible adjustments and prepare a return to work action plan. They must also participate, with the employee and treating doctor, in a meeting organized by the employee’s NAV counsellor within 26 weeks (“Dialogue meeting 2”) and also, if required, after approximately 9-11 months (“Dialogue meeting 3”). For the self-employed, NAV is responsible for organising Dialogue meetings 2 and 3, which involve the self-employed person and his/her doctor. 

Public social security and welfare provisions for salaried and self-employed workers in Norway are more generous than elsewhere [21]. Briefly, for salaried workers, sick leave is granted from day one for one year and the sick leave compensation benefit is equivalent to the regular salary [40]. After one year, the worker either transfers to a disability pension or receives a work assessment allowance (for a maximum of 3 years) or other benefits [41], equivalent to approximately 66% of their previous annual income. Self-employed people who do not have private insurance are granted sick leave compensation from day 17 for one year, at 66% of the past three years’ average income reported to the tax authorities. Although they may buy an insurance policy from the National Insurance Scheme to provide higher compensation if they become sick; only 11% do so [42]. After one year of sick leave, the regulations on work assessment allowance and disability pensions are more or less the same for self-employed people as for salaried workers.

### 2.2. Design

We used qualitative methods with thematic analysis inspired by Kvale and Brinkmann [43] and Graneheim and Lundman [44]. One-to-one semi-structured qualitative interviews were conducted with two participant groups: NAV counsellors and self-employed cancer survivors. The two groups were independent from each other, meaning that the counsellors did not talk specifically about the self-employed survivors included in this study, and vice versa. 

### 2.3. Participants

*NAV counsellors*: NAV counsellors were recruited by emailing local NAV offices and through personal contacts. To be eligible, counsellors had to, as part of their job, have experience with supporting self-employed workers and cancer survivors. Three participants were recruited through personal contacts and four from one local NAV office. One of the participants was known to the third author (BN). 

*Cancer survivors:* Study information was sent to personnel involved in cancer care in local municipalities, hospitals and labour and welfare administration offices in South-Eastern Norway, posted on the Facebook pages of the Norwegian Cancer Society, and distributed in two rehabilitations centres for cancer patients. We were interviewed on radio about the study. Eligible survivors had to have completed cancer treatment, not had a recurrence and have worked in their own business at the time of cancer diagnosis with their main income coming from this business. Ten cancer survivors contacted the researchers, of whom seven were eligible. Of the three who were not included, one had not been diagnosed with cancer and the two others did not want to or had time to be interviewed when we contacted them.

### 2.4. Interviews

All but one interview was face-to-face; the other interview, with a survivor, was by telephone. Interviews were at a time and location convenient for participants (mainly survivors’ homes and NAV counsellors’ workplaces). Participants were asked for their agreement to audio-recording and provided written informed consent. Interviews were guided by semi-structured interview guides. The guides for the two groups were not identical but covered broadly the same topics, including cancer trajectory, sick leave, work ability, self-employment and relevant measures to retain/return to work. In addition to some background questions regarding cancer and work, the interview guide for the self-employed cancer survivors was grouped into three parts: experiences related to treatment, health and work retention/return to work; factors and measures important to retain/return to work; and how the individual had experienced support from NAV. The opening questions for the last part were “How has the follow-up from NAV been for you?”, “How did you experience NAV’s focus on you and your company?” and “What (else) would you need/have liked from NAV?” The interview guide for the NAV counsellors was divided into five main parts: the typical trajectory for NAV clients; experiences with supporting self-employed people; experiences with supporting cancer survivors to retain/return to work; experiences with supporting self-employed cancer survivors. Examples of opening questions were: “What particular challenges do you see self-employed cancer survivors having?”; “Do you think you are able to help self-employed clients to keep the company afloat during treatment?”; “What kind of support does NAV offer that has been particularly successful for self-employed to retain/return to work?” Interviews evolved organically and specific questions asked and probes used varied from interview to interview. For both groups the interviews lasted approximately 45–75 min, were transcribed verbatim and anonymized. 

### 2.5. Analysis

The two interview sets were analysed separately, in conjunction with interview notes. A thematic approach was used [43]. Transcripts were read and re-read, and “meaning units” (i.e., words and statements that relate to the same central meaning through their content and context), were coded into a description close to the transcribed text. The condensed meaning units were organised into categories and subthemes. Themes were developed and similarities and differences between categories and subthemes were sought within and across transcripts and interview sets. Where there were links between interview sets, this has been highlighted. Direct quotes that illustrate participants’ narratives are provided. Each quote is followed by the relevant participant ID number and an indication of whether the interviewee was a survivor (“SE”) or counsellor (“NC”).

## 3. Results

Descriptions of the eligible seven self-employed cancer survivors and the seven NAV counsellors are respectively presented in Table 1 and Table 2. Six self-employed survivors were women, breast cancer was the most common diagnosis, and they varied in professional background. All the NAV counsellors had long experience with supporting cancer patients and self-employed people (mean = 21.4 years, range = 4–38 years) and they all had university level education. At time of the interview, the counsellors worked in a range of NAV departments. 

### 3.1. Self-Employed Cancer Survivors’ Experiences of Work After Cancer and with NAV

All self-employed cancer survivors said their business was very important to them, and that working a lot was a “lifestyle”. Although technically on sick leave almost all worked during and after cancer treatment (albeit mostly part-time and with reduced efficiency). They felt responsible for keeping the business running and securing an income for themselves and for their employees. All struggled from day one of the cancer diagnosis with worries about delivering services and products for customers, meeting ongoing expenses and future work ability. Related to the question on how they experienced the support from NAV, three themes emerged: (1) Poor support from NAV, (2) cheating the system, and (3) the need for private income protection insurance. 

#### 3.1.1. Poor Support from NAV

All survivors described receiving financial support from NAV (sick leave benefits) during the first year post-diagnosis. None was followed up by any NAV counsellor while on sick leave. Three received work assessment allowance after one year of sick leave; they described how NAV contacted them to formulate an activity plan for their return to work and that this was the first contact they had had from anyone in NAV. 

All survivors had been uncertain about how much sick leave benefit they would receive. They had expected that NAV would help them with “everything” related to their business. Some had phoned NAV but described how no-one could give them the information they needed. They perceived NAV as not caring about them.

*Of course, I would have appreciated receiving help from NAV, and them being attentive and helpful. But they didn’t care. They didn’t give a damn about me!*

(SE7)

*The disease gave me enough worries; I was hoping that NAV could give me a feeling of safety and predictability (but they did not).*

(SE2)

#### 3.1.2. Cheating the System

Survivors described how, while on full or part-time sick leave, they had to fill out NAV forms reporting how many hours they had worked, so that their sick leave benefit could be reduced accordingly. They explained that, because of treatment side effects (e.g., neuropathy, fatigue) they were not as efficient and work tasks took longer than usual, but that the system assumes that those working while on sick leave are as productive/effective as when they are well. Survivors described this system as unfair. Several reported to NAV that they had not worked when they actually had (which is illegal) saying that they did this because their financial situation was difficult. A poor financial situation was perceived as NAV not caring about them and thus linked to the “poor support from NAV” theme.

*I was sick listed the whole year and ticked off [on the form] that I didn’t work. I worked the whole year but no one from NAV contacted me during that year no meetings or telephone.*

(SE4)

*My work at the farm had to somehow be kept secret from NAV.*

(SE6)

To ‘cheat’ NAV seemed necessary because of financial hardships but at the same time, it evoked feelings of shame. 

#### 3.1.3. Need for Income Protection Insurance 

All survivors described how, before the cancer diagnosis, they were healthy and had plans for their future life and business. Few had reflected on what would happen if they got a serious disease. Few had previously bought the extra insurance policy from the National Insurance Scheme, but all had extra income protection insurance by the time of interview. As one survivor noted:

*You have to think of the worst-case scenario when you are self-employed. I didn’t do that and therefore I didn’t have any health insurance.*

(SE1)

### 3.2. NAV Counsellors’ Experiences of Supporting Self-Employed Cancer Survivors

Five themes emerged from the NAV counsellor interviews: (1) Survivors’ motivation to stay in work; (2) Taking the role of the employer; (3) Leaving self-employed cancer survivors in peace; (4) System and regulations; (5) Lack of resources and means in NAV.

#### 3.2.1. Survivors’ Motivation to Stay in Work 

The NAV counsellors’ descriptions of self-employed survivors’ working lives largely echoed those of survivors. They observed that the self-employed generally worked more hours/week than salaried workers and that this held during cancer treatment. Those who took sick leave were away from work for less time than salaried survivors. The counsellors considered that work was more important for self-employed, than salaried, survivors and that they were more motivated to continue working during treatment or return to work post-treatment.

*Many self-employed love their work. Therefore, the self-employed hold that they become healthier by working than “just being sick”.*

(NC5)

*The self-employed have an ownership of their work and then they push themselves. I would think that many push themselves longer than they would have done if they were ordinary employees. I think many of the self-employed [who have cancer] push to their limits before they throw in the towel.*

(NC1)

The counsellors were unclear whether self-employed survivors’ high work motivation was because work was so important to them or because of financial issues. They underlined that working during or after cancer treatment was important for the individual and for society, and observed that their own responsibility of supporting people to work was easy to discharge when people were as motivated as self-employed cancer survivors. However, some counsellors were unsure whether it was healthy to work as much as some self-employed cancer survivors did during and after treatment. 

#### 3.2.2. Taking the Role of the Employer 

The counsellors described the important role employers play in supporting return to work after cancer for salaried workers and observed that the lack of an employer meant that the situation for the self-employed could be more difficult and complex. They considered it important that they fulfilled the same role for self-employed cancer survivors as the employer did for salaried survivors.

*We must follow up the self-employed workers because they don’t have anyone to support them. Therefore, we substitute for the employer (that they do not have), and we are supposed to have consultations with them. We do the employer’s job.*

(NC5)

The counsellors also stressed the importance of their own engagement with each client, speaking about how it was important to provide both informative, instrumental and emotional support for different people. But, for cancer survivors, while they wanted to be supportive, they tried not to get too emotionally involved.

*Cancer is a terrible disease that hits the entire family and it is not easy to listen to all the histories about their children and what they are experiencing. One has to be supportive but at the same time professional.*

(NC5)

The counsellors described how they tried to provide self-employed cancer survivors with information to make decisions about work. They emphasized to survivors (both employees and self-employed) that it is important to stay engaged with work.

#### 3.2.3. Leaving Self-Employed Cancer Survivors in Peace

The counsellors spoke about cancer being “difficult” because of its sometimes long treatment trajectory and the risk of death; they recognised that patients can have a lot to deal with and return to work can take time. They considered it was inappropriate to pressurise survivors to return to work and this meant that they left survivors in peace (i.e., they did not contact them). They viewed this as showing good will and being considerate. They also noted that cancer survivors (compared to those with other medical conditions) were often keen to resume working. Therefore, they tended to prioritise groups with other conditions (e.g., mental or musculoskeletal conditions) who needed more encouragement to resume working.

*According to my opinion, if you have got cancer it is OK that you are allowed to be sick.*

(NC4)

*When it comes to cancer, we stay in the background. We follow them and see how it develops. We choose to lie low because the cancer patients have more than enough to deal with themselves. So, we don’t prioritize them.*

(NC1)

To leave the self-employed survivor in peace seems to be linked, but in a contradictory way, to the theme “taking role of the employer”, i.e., to leave survivors alone does not seem to provide informative, instrumental and emotional support as a substitute “employer”.

#### 3.2.4. System and Regulations 

The counsellors highlighted several aspects of the current regulations that were difficult for cancer survivors in general and often unfair for self-employed survivors in particular and said these made it more challenging for them to support self-employed survivors than it should be. For example, all counsellors noted that sickness benefit covers 100% of employees’ salary for one year then reduces to 66% of pre-illness income. Those with cancer often make the transition to the lower level of benefit because treatment and rehabilitation can take a long time. Those with a low income can find themselves in a particularly difficult situation. For self-employed the income is 66% from day one and the period with reduced income is therefore longer for them than for salaried.

*I have a client now who receive hormone therapy after breast cancer. She has received that treatment for two and a half years, and after two and a half years more, a new five year period of treatment will be considered. People get desperate over having such a low income while they are waiting to become cured.*

(NC1)

In addition, and in line with the self-employed survivors’ descriptions, the counsellors considered that that system of registering hours worked during sick leave and work allowance period was not fair for the self-employed since they often were not able, because of low work capacity, to generate as much income per worked hour as they could before the cancer treatment.

The counsellors described challenges for the self-employed resulting from fluctuating income. Because sickness benefits are based on mean income from the past three years registered in the taxation system, self-employed survivors may receive limited financial support.

*If you are running a business, you may have worked last year and got the income this year. It can also be the opposite that you work this year and get your income next year. Then you may come out of it very bad although they calculate the income from the last three years even if you really work a lot and earn good money at the moment you get sick listed.*

(NC6)

The counsellors observed that that the self-employed knew a lot about taxes, VAT and other duties and rights in normal working life, but that surprisingly few knew what benefits they were entitled to when becoming sick. All counsellors considered it important that the self-employed become better informed about their entitlements. Some felt it was the duty of public authorities to provide such information, but others considered it the responsibility of the self-employed themselves.

*People must first and foremost learn to check laws, regulations and their own rights. As a point of departure, we have a responsibility ourselves. It is too easy when the next day comes to say ‘what shall I do now’ or ‘what am I going to live from?’ It is too late when you are already sick.*

(NC2)

Some counsellors described the self-employed as “irresponsible”, reporting that few have income protection insurance should they become ill (which can be purchased from NAV). They also noted that self-employed people may take advantage of the taxation system for self-employed people when they are well, not considering that this may have negative effects for them if they subsequently become ill.

*They claim so much tax deductions and don’t ensure that they have enough income if they become sick. I don’t believe they even think that they can become sick. Suddenly you stand there and you have deducted so much tax that you “are in zero”. Then, you don’t have any working income to claim refunded! “So where did you find the money for living?” I have asked more than one client.*

(NC7)

#### 3.2.5. Lack of Resources and Means in NAV

The counsellors described having too many clients and stated that they could do a much better job in supporting people if they had fewer. They found it difficult to prioritise to whom they should provide more support than simply ensuring they get the benefits they are entitled to:

*I have 100 to 150 persons to follow up, so it is self-evident that I cannot follow up each and every with the same quality.*

(NC5)

Some said that the regulations in general are rather good but that some counsellors lack sufficient knowledge about them. Others claimed that some NAV counsellors do not put enough effort into finding the best solutions for cancer survivors and other clients, failing to apply the discretion available to them. Some spoke about a need for more competence and training in NAV for following up the self-employed in general and self-employed cancer survivors in particular, and/or more working time allocated to working specifically with self-employed.

*The regulations are of course not optimal, there is very little relating to the self-employed. They are so incredibly diverse, from big successful entrepreneurs who have started with two employees and now have 40 employees to one-man businesses and free-lancers. They are often in a difficult situation and they are often young, right! It is very difficult to make simple, specific, and just rules for such a diverse group, and in a system like NAV you are very dependent on that. Then, if those of us who work with the self-employed had more time and knowledge, and could go deeper into each and every case, it would probably be profitable for society.*

(NC1)

The counsellors noted that NAV itself and the wider system does not have any means to support the self-employed in maintaining the operation of their business while sick (linking also to the theme “System and Regulations”). They suggested NAV could, for instance, provide practical and financial support for replacement staff, guidance and support when negotiating with banks and creditors and/or financial support for re-launching the business after a pause because of the cancer treatment.

## 4. Discussion

One of the most important questions for newly diagnosed cancer patients is what support they are entitled to in the welfare system [8]. This seems particularly relevant for self-employed cancer survivors. While the self-employed in our study had received financial support from NAV, none had received any helpful support or guidance from NAV counsellors. 

Overall, the experiences of self-employed cancer survivors and NAV counsellors were rather similar. Both groups held that the self-employed (and, in particular, those with cancer) worked hard, were very engaged in their work and that they were motivated to retain or return to work quickly; the self-employed worked a lot during cancer treatment; self-employed people should in general be more aware of the need for extra insurance before becoming ill; financial issues were more complex for the self-employed than for salaried workers; and the self-employed got little support and attention from NAV the first year after a cancer diagnosis. The counsellors stated that they consciously left the self-employed cancer survivors in peace because they thought cancer patients should “be allowed to be sick” whereas the self-employed survivors interpreted this well-meaning inaction as neglect and a very poor service. Although the counsellors did not mention that self-employed survivors cheated the system by not reporting how much they worked while receiving sick-leave or work allowance benefits, they stated that the system was not fair for the self-employed because they were not as productive per hour worked as they were before cancer, and that, therefore, their income therefore was reduced. NAV counsellors gave in-depth information about the NAV system, and how limited resources/high work demands and inappropriate regulations and means were reasons for why it was difficult for counsellors to support appropriately the self-employed as a group.

Although the counsellors were very aware that they should follow-up and support the self-employed, our study suggests this does not appear to happen in practice: both the self-employed and the counsellors described little contact during or after cancer treatment. This echoes Becken, et al. [45] who found that NAV rarely holds the compulsory Dialog meeting 2 for cancer survivors, a meeting between employer (if relevant), employee, GP and NAV due before week 26 of sick leave. This may be because the counsellors (as they report here) do not want to pressure cancer survivors, but it means that self-employed survivors in particular who lack support from an employer and can have complex financial situations may be denied valuable support. It has been shown that cancer survivors are less satisfied with NAV services than other NAV clients [46], and Becken et al. [45] claim that the NAV system is particularly poorly designed for supporting self-employed people. Based on these studies and on our findings, it seems reasonable to conclude that self-employed people are more dissatisfied with NAV services compared to employees. 

Although the regulations require the self-employed to report how many hours they work during sick leave, the self-employed survivors in this study indicated that they did not report this correctly because they needed the sick leave benefits to manage financially during a time of low income. They were ashamed of cheating NAV and the public social services but felt they were forced to do this because the system did not make any allowance for their reduced productivity due to the cancer and/or their need to have an income. This adds to the shame they can experience around getting cancer and not being able to keep the business afloat [22]. The counsellors wanted to provide both emotional and practical support to the self-employed cancer survivors. In order to appropriately support survivors, it is important that NAV counsellors (and health professionals) are aware of, and sensitive to, the ways in which self-employed survivors may feel ashamed. 

The cancer trajectory can be long-lasting, and the one-year sick-leave limit allowed within the regulations may be too short for cancer survivors. Both the self-employed and the counsellors regarded this as a problem. A study more than a decade ago identified this as a problem [46], and a recent Norwegian official report [47] suggests the one-year limit should be extended for those who work part-time while sick the first year. Such a system may also motivate the self-employed to report how much they actually work during sick leave. 

Some counsellors considered the benefit entitlement for the self-employed unfair (i.e., lower level of sick leave benefit and basing the amount paid on an income that can be irregular). It has been documented that the definitions of what constitutes eligible income for the calculation of benefits varies between NAV departments [48] and therefore an individual’s income and, consequently, their benefits may be wrongly estimated. If this is the situation in general, it seems likely it will be worse for the self-employed, whose income is likely harder to estimate than that of someone with a regular monthly salary. 

One of the main aims of the 2006 NAV reform was to change the NAV employees’ role from a caseworker to a counsellor, meaning that cases should be closed by discretion, rather than following the rules [49]. Although our study indicates that there are idealistic counsellors fulfilling the aim of the NAV reform, some interviewees spoke about how discretionary powers were often not used. Others have also reported that there are few discretionary decisions or decisions adapted to meet applicants’ needs [34,50]. Not using discretion may adversely impact self-employed cancer survivors because of the complexity of their condition and financial situation, and lack of employer support. Counsellors observed that the self-employed were, in general, not well aware of their rights and entitlements on becoming sick. This suggests NAV could be more pro-active, seeking to inform the healthy self-employed about their (reduced) rights when becoming sick and encouraging them to acquire additional insurance. 

The counsellors also focused their own working situation: many clients, time/high job demands, low competence, and lack of means relevant for the self-employed and how that impacts the support they provide. Others have also shown that the NAV counsellors have a high work load [37,51] and have to set their own priorities in terms of which clients they support [33]. It was noteworthy that the counsellors reported both that they deliberately left self-employed cancer survivors in peace due to the challenging nature of cancer but, at the same time, they considered that it was more important to assist and guide other NAV client groups (e.g., those with mental health or addiction problems) back to work. In a review of accountability and delegated discretion in activation work in NAV Andreassen [33] concluded that NAV’s standards for follow-up which are more focused on the needs of the system rather than those of the clients may result in inadequate prioritisation of activities and clients [37,51]. Our findings indicate that self-employed cancer survivors are a group who are not prioritized.

Some of the counsellors wanted more training in guiding and supporting self-employed people. Such a claim has recently been supported by research suggesting that NAV counsellors need more training in general and in particular in relation to labour market issues [33] and the concepts and regulations underpinning the calculation of benefits [48]. It has been shown that structured training for NAV counsellors has improved the quality of their supervision of people having problems with taking part in working life [52] and there is no reason to believe that training related to the particular challenges of self-employed should not show similar positive results. 

### 4.1. Implications

The results of this study indicates that there are many measures that could be implemented to improve the NAV services for self-employed cancer survivors. First, it seems essential that NAV counsellors should have fewer clients and less work demands so that they have time to engage properly in the somewhat difficult situation of self-employed. This situation is characterized by not having a supportive employer to address problems while, at the same time, experiencing a more difficult and complex financial situation. Related to this, it seems that the counsellors need more education and training and/or support in dealing with the financial challenges self-employed often encounter. 

The NAV counsellors may also benefit from feedback about how self-employed people experience NAV, that is, that they feel neglected, and that leaving self-employed cancer survivors in peace is not a good way of supporting this group of clients.

The NAV system need to encourage NAV counsellors to use their discretionary powers more actively. This seems particularly necessary when supporting self-employed clients since their individual situations can be very different and their financial circumstances are so dependent on the kind of business they run, the number of employees they have, how the business is doing financially pre-diagnosis, and, how the labour market is in general.

More generally, NAV should be more proactive, seeking to inform healthy self-employed people about their (reduced) rights when becoming sick and encouraging them to acquire additional insurance.

NAV needs to develop and offer more business-related means particularly relevant for self-employed people. Examples of such measures may include financial support for employing a substitute worker, financial support to re-launch a business after a period of illness, and to support cancer survivors to negotiate with banks and creditors.

The system of sick leave benefits for one year only, irrespective of being partly in work or not, should be revised for people with severe disorders with long treatment trajectories. This would be of particular help for self-employed cancer survivors. Also, the system of registering number of worked hours during sick leave should be revised for self-employed people because they are not able to generate enough income during worked hours because of low work capacity. 

Overall, there is a lack of intervention research on cancer survivors and work [16,17] and to our knowledge, there is no research that has investigated the effects of such measures mentioned above (or, indeed, any other interventions) on the challenges self-employed survivors experience in keeping their business afloat. Research on this topic is urgently required both in Norway and other countries.

Compared to Norway, in most other countries self-employed people have less support and fewer rights and entitlements when ill [21]. This suggests that the experiences of self-employed cancer survivors in other countries may be even worse than those reported here. In addition, the challenges in supporting self-employed survivors experienced by the NAV counsellors are probably present in other welfare systems across Europe (and beyond). However, as far as we are aware there is no other published work on this topic. Therefore, research is required to explore experiences of both self-employed survivors and social welfare officers in other countries with different welfare systems than the Norwegian system. 

### 4.2. Methodological Limitations

Since very little is known about self-employed cancer survivors’ experiences with the social welfare system and counsellors’ experiences with supporting self-employed individuals, we chose to use qualitative semi-structured interviews with an open and inductive approach to reveal as many aspects as possible of the survivors’ and counsellors’ experiences. The study included self-selected and relatively few informants from each group. Although representativeness of participants is not as important in qualitative research as it is in quantitative studies, this may restrict the study’s trustworthiness. In the current study, only one male survivor and one male NAV counsellor participated. In the cancer survivorship literature female gender has been associated with a lower rate of return to work after cancer [53]. The explanations for this are unclear but may include societal and cultural attitudes towards work among men and women [54]. Thus, the fact that most of the survivors in this study were female may be a factor in the experiences they reported. Further, the few informants precluded us being able to explore whether there were particular characteristics of participants that were associated with different experiences (such as whether male informants responded differently from females or whether those who had been counsellors for longer felt better able to support self-employed survivors). 

Another limitation is that four of the seven NAV counsellors were recruited from the same NAV office. This may also have affected the counsellors’ experiences and opinions. Nevertheless, the data revealed new and nuanced information about challenges to support self-employed people with cancer, and it is the same training, regulations, and means all NAV counsellors must adhere to within the bigger NAV system. Although the self-employed survivors and counsellors were not dyads, we consider the presentation of experiences from both self-employed and NAV counsellors as a strength of the study as it gives a fuller picture of the investigated issue. The results of this first qualitative study on supporting self-employed cancer survivors within the social welfare system may be used as a baseline understanding for other researchers and practitioners who want to investigate, understand, and improve social welfare services for self-employed cancer survivors in other settings. Further research using both qualitative and quantitative research methods is clearly needed.

## 5. Conclusions

The findings of the present study suggest that the social welfare system of Norway is not adapted to support sick self-employed people appropriately. Improvements must be implemented related to the legal rights of self-employed, means of addressing self-employed individuals’ need to keep their business afloat during and after treatment, and NAV counsellors’ high work demands. To increase the NAV counsellors’ knowledge about the difficult situation self-employed people experience when diagnosed with cancer seems necessary for the counsellors to use their discretionary powers appropriately. 

## Figures and Tables

**Table 1:** Self-employed cancer survivors’ characteristics [22]. Copyright 2021, with permission from Springer Nature.

Informant	Sex	Cancer Diagnosis	Profession (Enterprise Type ^1^)	Employees	Treatment Duration (Months)	Worked During Treatment	In Work at Interview
SE1	F	Breast	Chiropractor (SP)	0	5	Yes	Yes, 100%
SE2	M	Lung	Shop owner (Ltd.)	2 (part-time)	12	Yes	Partly
SE3	F	Breast	Hair dresser (Ltd.)	4 (part-time)	7	Yes	Partly
SE4	F	Breast	Shop owner (Ltd.)	2 (part-time)	10	Yes	Partly
SE5	F	Lymphoma	Chiropodist (SP)	0	4	No	No
SE6	F	Breast	Farmer (SP)	1	12	Yes	Partly
SE7	F	Breast	Artist (SP)	0	9	Yes	Yes, 100%

^1^ S = Sole Proprietor, Ltd. = Limited Company.

**Table 2:** NAV counsellors’ characteristics [22]. Copyright 2021, with permission from Springer Nature.

Informant	Sex	Years of Higher Education ^1^	Years as NAV Counsellor	Current Work Tasks/NAV Department
NC1	F	3	4	Work assessment allowance
NC2	F	1	38	Sick leave
NC3	M	5	7	Labour market
NC4	F	2	36	Disability and work assessment allowance
NC5	F	3	11	Sick leave without employer
NC6	F	3	16	Sick leave without employer
NC7 ^2^	F	2	38	Experience from all departments in NAV

^1^ post-high school; ^2^ Recently retired.

## Data Availability

The study was based on qualitative data, which are stored according to instructions from the Regional Ethics Committee, South-Eastern Norway. The data cannot be accessed by any other than the researchers involved in the current study.
